# Preferences of Individuals With Obesity for Online Medical Consultation in Different Demand Scenarios: Discrete Choice Experiments

**DOI:** 10.2196/53140

**Published:** 2024-11-27

**Authors:** Yaolin Hu, Jian Wang, Jianbo Zhou, Yuanyuan Gu, Stephen Nicholas, Elizabeth Maitland

**Affiliations:** 1 School of Economics Peking University Beijing China; 2 Dong Fureng Institute of Economic and Social Development Wuhan University Wuhan China; 3 Macquarie University Centre for the Health Economy Macquarie Business School & Australian Institute of Health Innovation Macquarie University Sydney Australia; 4 Health Services Research and Workforce Innovation Centre Newcastle Business School University of Newcastle Newcastle Australia; 5 Australian National Institute of Management and Commerce Sydney Australia; 6 School of Management University of Liverpool Liverpool United Kingdom

**Keywords:** online medical consultation, obesity, discrete choice experiments, telehealth, telemedicine

## Abstract

**Background:**

Obesity is a unique chronic disease, with China having the largest number of people living with overweight and obesity in the world. There has been little research from the demand perspective for online medical consultation (OMC) by individuals living with obesity. With the growing demand for obesity OMC, especially due to the emergence of new pharmacotherapies, such as glucagon-like peptide-1 receptor agonists, individuals living with obesity are seeking both advice on obesity management and the prescription of obesity drugs. Therefore, our demand scenarios defined 2 OMC motivations to manage obesity: “For-Drugs” use and “For-Advice” use.

**Objective:**

This study aims to assess and compare the preferences for For-Drugs and For-Advice OMC among individuals living with obesity in China.

**Methods:**

Following the International Society for Pharmacoeconomics and Outcomes Research’s checklist and comprising 400 participants assigned to the For-Drugs scenario and 400 to the For-Advice scenario, the For-Drugs and For-Advice preferences were estimated through discrete choice experiments. The groups in the 2 scenarios followed a similar distribution, and the 2 different demand scenarios shared the same discrete choice experiment design, comprising 16 choice sets with 6 representative attributes. Mixed logit modeling was used to estimate the willingness to pay and relative importance scores.

**Results:**

Doctors with well-known and general expert titles, versus ordinary doctors; doctors from high-level, provincial, tertiary, and municipal hospitals, versus lower-level county hospitals; less waiting time; and lower OMC fees were preferred in both the For-Drugs and For-Advice scenarios. The differences between the 2 scenarios lay in the consultation format, consultation duration, and the relative importance of consultation duration versus waiting time. The For-Advice group preferred telephone consultations, while the For-Drugs group did not; the For-Drugs group preferred longer consultation duration (β=.029), while the For-Advice group preferred shorter consultation duration (β=–.030); and the For-Drugs group rated consultation duration higher than waiting time, while the For-Advice group rated the waiting time as more important than consultation duration. Combined with our qualitative research, the differences can be explained by the different consultation needs in the 2 scenarios, where longer patient consultations were preferred by the For-Drugs patients who sought detailed advice on drug side effects, while quick and direct responses were preferred by the For-Advice participants.

**Conclusions:**

By revealing user preferences on costs, doctors’ titles and hospital level, wait time, and consultation duration and format, our research informs OMC platforms, OMC regulators, and doctors on market segmentation and service differentiation strategies.

## Introduction

### Seeking Web-Based Obesity Consultation

Obesity is a global public health problem [1], and China has the largest number of people living with overweight and obesity in the world [2]. With individuals living with overweight and obesity increasingly seeking medical support, there is an urgent need to better understand and improve obesity management in China, including the adoption of electronic health technologies [3]. In China’s telemedicine industries, online medical consultation (OMC) is one of the most recognized and accepted web-based medical services. Promoted during the COVID-19 pandemic, OMC offers private and confidential communication, which can help relieve stigma and other stress burdens on individuals living with obesity and offer increased obesity treatment adherence relative to offline consultations [4,5]. OMC also reduces offline medical consultation costs, such as transportation and forgone work time costs [6].

OMC is also popular for its convenient prescriptions [7], a feature especially appealing to individuals living with obesity. Not only is obesity a chronic disease, but it is also correlated with comorbidities, such as diabetes, heart disease, hypertension, and cancer [8], which means individuals living with obesity have a high demand for multiple long-term medications. OMC prescriptions offer significantly time-saving experiences for individuals living with overweight or obesity who previously could only refill their medicines at hospitals. In China, OMC regulations allow patients to buy medications through web-based platforms after providing evidence of a prescription.

Recently, web-based obesity medicine prescriptions have gained a new level of popularity in China. Promising findings of glucagon-like peptide-1 receptor agonists, such as semaglutide and beinaglutide [9,10], have spurred many individuals living with obesity to adopt new pharmacotherapies, leading this group to increasingly access OMC to get prescriptions for weight-loss drugs. This new demand by individuals living with obesity has driven some Chinese OMC providers to channel patients into different consultation modes, such as “For-Advice” and “For-Drugs.” The demand for the latter is so large that some OMC platforms have become increasingly dependent on digital medication sales [11,12].

In this study, we assess the preferences for OMC among Chinese adults living with obesity. Given there are 2 different motivations for OMC, we set out 2 distinct demand scenarios—“For-Advice” and “For-Drugs”; designed 2 discrete choice experiments (DCEs) to elicit consumer preferences under each scenario; and compared the “For-Advice” and “For-Drugs” groups [3]. This study provides new insights into the future development of OMC for telehealth providers, doctors, and regulators [12].

### Research Motivation

Previous studies on China’s OMC failed to distinguish between different diseases, with any results subject to selection bias [13,14], and failed to distinguish between different types of demand, such as For-Advice and For-Drugs [15]. By focusing on the needs of individuals living with overweight and obesity for OMC, more detailed findings from the demand perspective can be revealed. Focusing on the preferences of individuals with obesity for OMC also avoids disease selection bias and provides disease-specific advice for medical industry organizations and public health regulators [16,17].

## Methods

### Ethical Considerations

The experiments were ethically reviewed, and the study was approved by the Wuhan University, Faculty of Dong Fureng Institute of Economic and Social Development, Research Ethics Committee (dfr202201). The study was conducted according to the principles established by the Declaration of Helsinki, and data were anonymized. Participants were provided with information regarding the study and asked to sign an informed consent form before taking part. Participants who completed the experiment received compensation.

### Definition of Different Demand Scenarios

We used DCE, a widely used method in health economics research, to elicit the preferences of individuals living with obesity [18,19]. With the help of the community workers from the community health centers in Wuhan, we undertook a 1-hour focus group discussion with 3 individuals living with overweight or obesity in July 2021 by using nonprobability convenience sampling. Facilitated by the corresponding author, the 3 interviewees in their 20s, 40s, and 50s discussed their obesity management aims, OMC experience, OMC costs, and OMC versus offline treatments. The focus group discussion confirmed our hypothesis of 2 different OMC demand scenarios (For-Drugs and For-Advice), which had been neglected by previous research.

After the focus group discussions, we consulted 6 obesity experts on OMC, obesity treatments, and DCE design through telecommunication in July 2021, comprising 2 industry experts in obesity treatment, 2 industry experts in OMC, and 2 academic professors focused on telemedicine.

Finally, we conducted 2 pilot surveys in Wuhan from July 2021 to August 2021. With the help of Wuhan’s community health workers, we selected individuals living with obesity who visited the health center by using nonprobability convenience sampling. First, 20 individuals living with overweight or obesity assessed the accuracy and appropriateness of the questionnaires and scenarios. After receiving the feedback from the first pilot survey, we modified the questionnaires and undertook the second pilot survey with another 20 individuals living with overweight or obesity as a final check.

The “For-Drugs” OMC demand scenario represents the case where OMC is for a prescription for specific weight-loss drugs or other drugs for obesity-related comorbidities. Based on the results of our focus group study and pilot surveys, this demand scenario was confirmed for 3 reasons. First, the purchase of medicines is one of the most demanded services in outpatient hospitals. Second, patients usually incur offline transportation, consultation waiting time, and lost work income costs to update prescriptions in hospitals. Third, most OMC platforms in China provide pharmaceutical e-commerce services, with some platforms mainly providing pharmaceutical e-commerce for drug sales as their profit motive rather than consultation services. For adults living with overweight or obesity, high-frequent medication demand via OMC is mainly supported by 2 motivations. The first motivation is to lose weight where doctors’ prescriptions are required. The second motivation is to acquire long-term medications to treat other obesity-related chronic diseases, such as hypertension, diabetes, and hyperlipidemia. Complying with the legal requirements for web-based drug purchasing in China, the final description of the “For-Drugs” demand scenario is shown in Multimedia Appendix 1.

The second “For-Advice” demand scenario represents the case where OMC is for medical advice only. One of the reasons why the Chinese government promotes OMC has been to allocate scarce medical resources more optimally and allow people living remotely to access high-quality medical services. Further, losing weight is a long process, requiring frequent consultation on health management, diet, and exercise [20], which OMC can provide at a low cost. OMC also provides a high level of privacy, helping to overcome psychological barriers and poor treatment compliance for patients living with obesity. For these reasons, seeking medical advice is a major reason for using OMC. The final description of the “For-Advice” demand scenario is shown in Multimedia Appendix 2.

The final DCE surveys were conducted via a web-based platform, mainly based on 2 reasons. First, research suggests that in some situations, well-designed digital DCEs can better elicit respondents’ preferences compared with offline experiments [21,22]. Second, during our offline pilot surveys, we found that respondents living with overweight or obesity felt pressure and stigma to talk freely about their true preferences.

**Figure 1:**
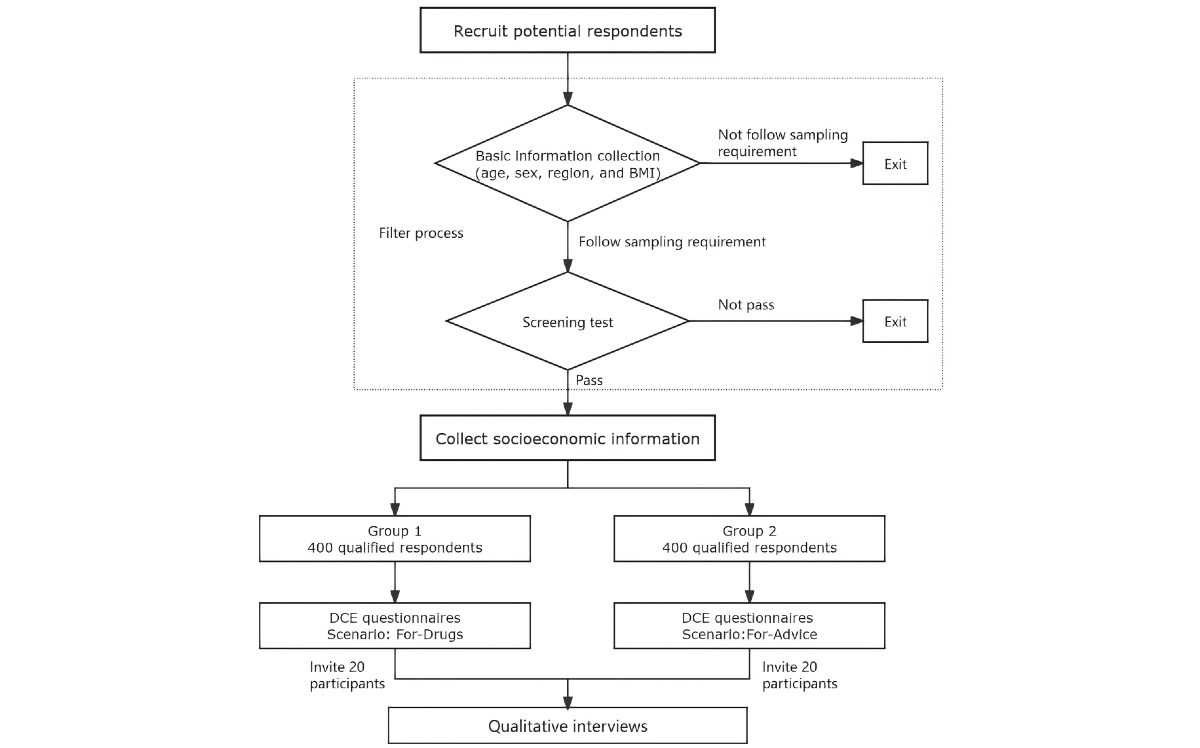
Flowchart of our web-based survey. DCE: discrete choice experiment.

As shown in Figure 1, we assigned respondents to group 1 (“For-Drugs” scenario) and group 2 (“For-Advice” scenario). The assignment of every respondent to different scenarios followed the same sample frame to ensure the 2 groups of respondents followed the same distribution characteristics of the obesity epidemic population in China. Respondents were not allowed to respond to both demand scenarios [23,24]. In our pilot surveys, respondents felt completing 2 DCEs under 2 scenarios were overwhelming and often got confused when they needed to imagine they were in 2 sets of scenarios [25].

### Sample Size and Data Collection

Obesity treatment involves both individuals living with overweight or obesity. We used the standards for adults living with overweight or obesity defined by China’s National Health Commission, with adults living with overweight having 24≤BMI<28 and adults living with obesity having BMI≥28 [26]. Based on the prevalent rate of overweight and obesity in China [27-29] and the sample size calculation formula [30]:

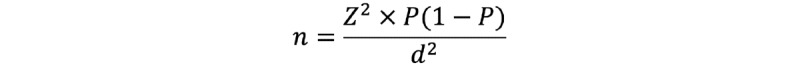



where *n* is the sample size; *Z* is the *Z* statistic for a level of confidence (1.96 for 95% CI); *P* is the expected prevalence; and *d* is precision, we set the precision at 0.1 *P*, which yielded a minimum national sample size of 400 individuals living with overweight or obesity.

We used quota sampling to recruit participants, with the prevalent rates of age, sex, and regions of participants defining the quotas in our survey [31]. The proportion of overweight and obesity in Chinese adults was derived from the National Report on Nutrition and Chronic Diseases in Chinese Residents [27] while the survey of 441 thousand adults by Zhang et al [28] provided the sex, age, and regional distribution parameters for adults living with overweight or obesity. To ensure the representativeness and quality of our sample, we designed a filter process before the experiments to recruit 800 qualified participants.

The filter process had 2 steps (Figure 1). The first step was to collect information on respondents’ age, sex, region, and BMI to ensure the participants recruited would follow the epidemiological distribution characteristics of obesity in China. BMI is a vital variable in our survey. In the pilot survey, we found that many potential participants did not know the meaning of BMI but knew their height and weight. We therefore collected participants’ height and weight information, automatically calculating participants’ BMI through our web-based experiment platform. Contracting with a commercial medical research firm with extensive contacts with doctors and hospitals across China, 2000 potential participants meeting the first filtering step were recruited. By email, phone, and in-person interviews, the commercial research firm verified the authenticity of the participants’ information with the patients and their doctors. The second step was a screening test to check if the potential participants understood the DCE choice tasks as intended. Those who failed the test were screened out. Through the screening test, the academic research team selected 800 participants.

### DCE Design

**Figure 2:**
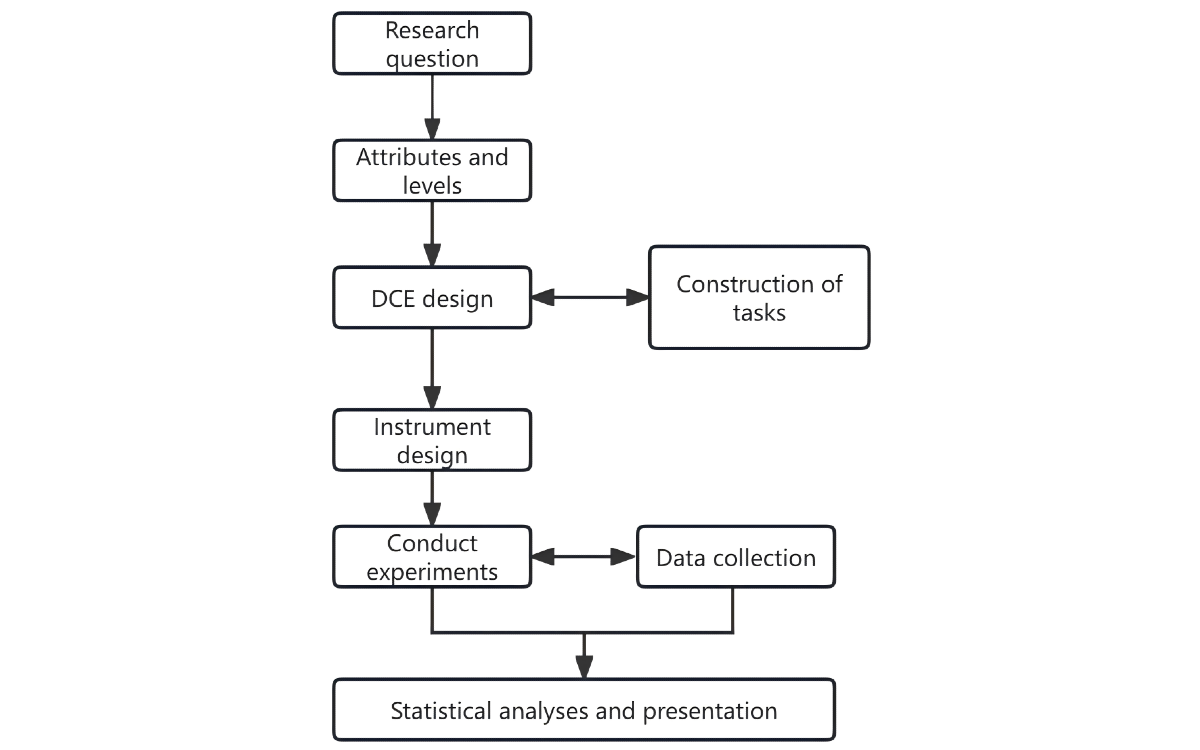
Processes of DCEs following the checklist recommended by ISPOR (International Society for Pharmacoeconomics and Outcomes Research). DCE: discrete choice experiment.

As shown in Figure 2, we conducted our DCE following the practice recommended by the International Society for Pharmacoeconomics and Outcomes Research (ISPOR) [19]; the checklist is shown in Multimedia Appendix 3.

The DCE attributes and levels were developed through a literature search, qualitative research on China’s OMC [12], and focus groups involving the OMC industry and academic professionals. We also undertook 2 rounds of pilot study to test the attributes and levels. The DCE attributes and levels are listed in Table 1.

**Table 1:** Attributes and levels in the DCE^a^ survey.

Attributes	Levels
Doctor level	Ordinary doctors General experts Well-known experts
Hospital level	County-level hospitals Municipal-level hospitals Provincial tertiary hospitals
Out-of-pocket cost (RMB^b^)	25 50 80 150
Waiting time (minutes)	15 30 60 180
Consultation format	Text consultation Telephone consultation
Consultation duration (minutes)	10 15 20

^a^DCE: discrete choice experiment.

^b^The conversion rate is approximated at 1 RMB ≈ US $0.14 on September 27, 2024.

The description of doctor levels and hospital levels uses the common terminology among the population and follows the regulations of China’s hospital management. The professional description of Chinese doctors’ titles is resident physician, attending physician, deputy chief physician, and chief physician. The higher the title, the higher the experience. However, in the pilot experiments, we found participants could not clearly define the differences between titles. We revised the description of the doctors’ level by using ordinary doctors (equal to resident physicians and attending physicians), general experts (deputy chief physicians), and well-known experts (chief physicians). In our web-based support, we provided an explanation of doctors’ titles. A similar process was also applicable to the confirmation of the description of the hospital levels. After searching the obesity OMC platforms, we found there were few doctors providing video obesity consultation, which may be explained by the stigma around obesity treatment. Text consultation and telephone consultation were the 2 consultation formats almost all the web-based doctors provided. In text consultation, users could send text, pictures, and voice messages to doctors and the doctors could respond later. Telephone consultation was more direct. Using the telephone system on OMC platforms, doctors accepted a user’s consultation request and then directly responded to the user [12].

We constructed the choice sets using Ngene software (ChoiceMetrics) and a D-efficient design was used to generate 16 choice sets, divided into For-Drugs and For-Advice participants. Each choice set was composed of 2 options with 1 more opt-out option using a dual-response design [32].

To assist participants, we provided easily available links to explanations of the attributes and levels in the web-based survey and our survey team also offered offline support. To encourage participants to complete the survey, only those completing the experiment were remunerated. We also designed the digital experiment to identify potential invalid responses. For example, when respondents chose the same choice option 3 times in a row, the digital experiment automatically reminded the respondents to make choices consistent with their true preference.

After the web-based DCE, we invited 5% (n=40) of our participants to take part in qualitative interviews. We mainly invited 2 categories of participants, one with typical responses and one with diverse choices. The 40 qualitative interviewees had representative characteristics in terms of age, region, and socioeconomic characteristics. Since the participants were distributed across China, we used telecommunication to interview the participants one by one. Each interview was completed with one interviewer asking questions based on the interview questionnaire and the other interviewer taking notes. Every interview lasted at least 10 minutes.

### Statistical Analysis

Based on random utility theory [31], the data were analyzed in a mixed logit (ML) model [33], wherein the utility that respondent *i* obtained from choosing alternative *j* in the choice set *s* is given by *U_isj_* = *V_isj_* + *ε_isj_* = *β_i_X_isj_* + *ε_isj_* where vector *V_isj_* is the observable systematic part and equals vector *β_i_X_isj_*, with vector *X_isj_* representing the alternative specific constant (ASC) and attributes of alternative *j* and vector *β_i_* representing associated preference parameters. The ASC represents preferences that are inherent and independent of specific attribute values. Vector *ε_isj_* is the unobservable random component and is independently and identically distributed as a type 1 extreme value [34]. The willingness to pay (WTP) [35] and relative importance scores (RIS) [36] were also computed based on the ML model. All the analyses were conducted with STATA 16 (StataCorp).

### Qualitative Analysis

Involving 40 DCE participants, the postexperiment qualitative interviews explored the participants’ feelings about the experiment, their preferences for and opinions about OMC, and their views about the different scenarios. One interviewer took notes, which were reviewed by the corresponding author, the note-taking interviewer, and one member of the academic research team to develop major themes and issues. The main themes and issues identified by each note assessor were compared and discussed.

## Results

Table 2 shows the participants’ socioeconomic information, including marital status, education, and income. Even though we used BMI, age, sex, and region to construct our representative samples [28], the distribution of marital status, education, and income between the 2 groups was also broadly comparable.

Table 3 shows the results of preferences and WTP space based on the ML model for the For-Drugs scenario and Table 4 shows the results for the For-Advice scenario. Similar preferences were found between the 2 scenarios. Participants in both scenarios preferred doctors with higher titles and from higher-level hospitals. However, the coefficients in the For-Advice scenario were higher than the For-Drugs scenario coefficients, implying For-Advice participants rated the requirements and their expectations of doctors higher than the For-Drugs participants when seeking professional advice. As for the consultation format, participants in the For-Advice scenario had a significantly higher preference for telephone consultations over text consultations (*P*=.002). Shorter waiting time and lower OMC fees were preferred in both scenarios. For-Drugs participants preferred longer consultation time (β=.029; *P*<.001), while For-Advice participants preferred shorter consultation time (β=–.030; *P*=.001).

Table 5 compares the differences in the 2 scenarios directly. Participants in the For-Advice scenario were willing to pay more for doctors with a higher title; doctors from higher-level hospitals; and RMB 9 (the conversion rate is approximated at 1 RMB ≈ US $0.14 on September 27, 2024) more for telephone consultation than text consultation. Participants in both scenarios were not WTP more for a longer consultation duration. Relative importance scores were also calculated based on the ML model, and the 3 most influential attributes were cost, hospital level, and doctor level in descending order, with costs accounting for more than 48% of the explanatory power in both scenarios. For For-Drugs participants, the importance of consultation duration was greater than waiting time, but For-Advice participants rated waiting time as more important than consultation duration. RIS estimation also revealed that in the For-Drugs scenario, the importance of the consultation form did not contribute to their choice but was significantly important (β=.029; *P*<.001) for the For-Advice participants.

Based on our post-DCE interviews with 40 participants, our qualitative feedback confirmed the conclusions drawn from the statistical analysis. Four main themes were identified: For-Advice and For-Drugs represented 2 distinct reasons individuals living with obesity selected OMC; the level of the hospital was more important than the title of the doctor; slow responses from web-based doctors can reduce users’ willingness to use and pay for OMC; and longer patient consultations were preferred by the For-Drugs patients who sought detailed advice on drug side effects, while quick and direct responses were preferred by the For-Advice participants.

**Table 2:** Characteristics of participants following distribution characteristics of obesity epidemic in China.

Variable				Overweight distribution (24≤BMI<28; n=271), n (%)		Obesity distribution (BMI≥28; n=129), n (%)		Overall distribution (n=400), n (%)
**Scenario: For-Drugs**								
	**Sex**							
		Male	145 (53.51)		63 (48.84)		208 (52)	
		Female	126 (46.49)		66 (51.16)		192 (48)	
	**Age group (years)**							
		18-34	46 (16.97)		25 (19.38)		71 (17.75)	
		35-54	96 (35.42)		45 (34.88)		141 (35.25)	
		55-74	93 (34.32)		44 (34.11)		137 (34.25)	
		75 and older	36 (13.28)		15 (11.63)		51 (12.75)	
	**Region**							
		North China	46 (16.97)		28 (21.71)		74 (18.50)	
		Northeast China	45 (16.61)		25 (19.38)		70 (17.50)	
		East China	38 (14.02)		15 (11.63)		53 (13.25)	
		Central China	36 (13.28)		19 (14.73)		55 (13.75)	
		South China	30 (11.07)		6 (4.65)		36 (9)	
		Southwest China	37 (13.65)		17 (13.18)		54 (13.50)	
		Northwest China	39 (14.39)		19 (14.73)		58 (14.50)	
	**Marital status**							
		Married	226 (83.39)		103 (79.84)		329 (82.25)	
		Not married	45 (16.61)		26 (20.16)		71 (17.75)	
	**Highest education**							
		Primary and below	23 (8.49)		10 (7.75)		33 (8.25)	
		Junior high	60 (22.14)		30 (23.26)		90 (22.50)	
		Senior high	35 (12.92)		19 (14.73)		54 (13.50)	
		Junior college	41 (15.13)		26 (20.16)		67 (16.75)	
		University	83 (30.63)		34 (26.36)		117 (29.25)	
		Master’s or doctorate degree	29 (10.70)		10 (7.75)		39 (9.75)	
	**Monthly income before tax (RMB^a^)**							
		≤2000	26 (9.59)		20 (15.50)		46 (11.50)	
		2001-6000	116 (42.80)		47 (36.43)		163 (40.75)	
		6001-12,000	100 (36.90)		54 (41.86)		154 (38.50)	
		12,001-35,000	26 (9.59)		7 (5.43)		33 (8.25)	
		≥35,001	3 (1.11)		1 (0.78)		4 (1)	
**Scenario: For-Advice**								
	**Sex**							
		Male	145 (53.51)		63 (48.84)		208 (52)	
		Female	126 (46.49)		66 (51.16)		192 (48)	
	**Age group (years)**							
		18-34	46 (16.97)		24 (18.60)		70 (17.50)	
		35-54	98 (36.16)		46 (35.66)		144 (36)	
		55-74	93 (34.32)		44 (34.11)		137 (34.25)	
		75 and older	34 (12.55)		15 (11.63)		49 (12.25)	
	**Region**							
		North China	46 (16.97)		30 (23.26)		76 (19)	
		Northeast China	45 (16.61)		25 (19.38)		70 (17.50)	
		East China	38 (14.02)		15 (11.63)		53 (13.25)	
		Central China	35 (12.92)		19 (14.73)		54 (13.50)	
		South China	32 (11.81)		10 (7.75)		42 (10.50)	
		Southwest China	37 (13.65)		15 (11.63)		52 (13)	
		Northwest China	38 (14.02)		15 (11.63)		53 (13.25)	
	**Marital status**							
		Married	234 (86.35)		114 (88.37)		348 (87)	
		Not married	37 (13.65)		15 (11.63)		52 (13)	
	**Highest education**							
		Primary and below	15 (5.54)		10 (7.75)		25 (6.25)	
		Junior high	74 (27.31)		33 (25.58)		107 (26.75)	
		Senior high	45 (16.61)		18 (13.95)		63 (15.75)	
		Junior college	51 (18.82)		23 (17.83)		74 (18.50)	
		University	64 (23.62)		37 (28.68)		101 (25.25)	
		Master’s or doctorate degree	22 (8.12)		8 (6.20)		30 (7.50)	
	**Monthly income before tax (RMB)**							
		≤2000	24 (8.86)		19 (14.73)		43 (10.75)	
		2001-6000	129 (47.60)		59 (45.74)		188 (47)	
		6001-12,000	91 (33.58)		39 (30.23)		130 (32.50)	
		12,001-35,000	20 (7.38)		11 (8.53)		31 (7.75)	
		≥35,001	7 (2.58)		1 (0.78)		8 (2)	

^a^The conversion rate is approximated at 1 RMB ≈ US $0.14 on September 27, 2024.

**Table 3:** Preference and WTP^a^ space for OMC^b^ in the For-Drugs scenario.

ASC^c^ or attribute and levels				Coefficient									SD						
			β				*P* value		95% CI		β			*P* value		95% CI			
**Preference estimates**																			
	**ASC**		2.994				<.001		2.323-3.665		3.177			<.001		2.400-3.954			
	**Doctor level**																		
		Ordinary doctors			Reference		Reference		Reference		Reference			Reference		Reference			
		Well-known experts			1.230		<.001		1.044-1.417		.565			<.001		0.278-0.853			
		General experts			.396		<.001		0.246-0.546		.225			.35		0.247-0.697			
	**Hospital level**																		
		County-level hospitals			Reference		Reference		Reference		Reference			Reference		Reference			
		Provincial tertiary hospitals			1.464		<.001		1.258-1.671		.944			<.001		0.693-1.195			
		Municipal-level hospitals			.946		<.001		0.770-1.122		.557			.001		0.237-0.876			
	**Consultation format**																		
		Text consultation			Reference		Reference		Reference		Reference			Reference		Reference			
		Telephone consultation			.131		.06		–0.004 to 0.265		.161			.54		0.674-0.353			
	**Consultation duration**					.029		<.001		0.013-0.045		.005			.80		0.034-0.044		
	**Waiting time**					–.003		<.001		–0.005 to –0.002		.016			.054		0.000-0.032		
	**Out-of-pocket cost**					–.026		<.001		–0.041 to –0.012		.046			.14		0.015-0.106		
**WTP space estimates**																			
	**ASC**		178.159				<.001		138.117-218.201		192.313			<.001		228.542-156.084			
	**Doctor level**																		
		Ordinary doctors			Reference		Reference		Reference		Reference			Reference		Reference			
		Well-known experts			67.127		<.001		56.573-77.681		28.927			<.001		44.883-12.970			
		General experts			18.496		<.001		9.049-27.943		1.640			.89		21.107-24.387			
	**Hospital level**																		
		County-level hospitals			Reference		Reference		Reference		Reference			Reference		Reference			
		Provincial tertiary hospitals			86.542		<.001		74.972-98.112		52.905			<.001		39.701-66.108			
		Municipal-level hospitals			57.800		<.001		47.169-68.432		28.406			.009		49.696-7.117			
	**Consultation format**																		
		Text consultation			Reference		Reference		Reference		Reference			Reference		Reference			
		Telephone consultation			6.556		.11		–1.452 to 14.564		8.485			.63		25.988-42.957			
	**Consultation duration**					–1.105		.26		–3.012 to 0.802		1.709			<.001		0.856-2.563		
	**Waiting time**					–3.642		<.001		–4.918 to –2.366		2.462			<.001		1.838-3.086		

^a^WTP: willingness to pay.

^b^OMC: online medical consultation.

^c^ASC: alternative specific constant.

**Table 4:** Preference and WTP^a^ space for OMC^b^ in the For-Advice scenario.

ASC^c^ or attribute and levels			Coefficient							SD				
			β		*P* value		95% CI		β			*P* value		95% CI
**Preference estimates**														
	**ASC**		3.106		<.001		2.426-3.786		3.080			<.001		2.445-3.714
	**Doctor level**													
		Ordinary doctors	Reference	Reference		Reference		Reference			Reference		Reference	
		Well-known experts	1.362	<.001		1.142-1.582		.969			<.001		0.697-1.241	
		General experts	.418	<.001		0.249-0.587		.612			<.001		0.313-0.912	
	**Hospital level**													
		County-level hospitals	Reference	Reference		Reference		Reference			Reference		Reference	
		Provincial tertiary hospitals	1.549	<.001		1.309-1.789		1.269			<.001		0.992-1.546	
		Municipal-level hospitals	1.095	<.001		0.905-1.285		.471			.02		0.083-0.858	
	**Consultation format**													
		Text consultation	Reference	Reference		Reference		Reference			Reference		Reference	
		Telephone consultation	.234	.002		0.084-0.384		.322			.20		0.169-0.813	
	**Consultation duration**		–.030		.001		–0.047 to –0.013		.005			.78		0.030-0.040
	**Waiting time**		–.004		*<*.001		–0.005 to –0.003		.010			<.001		0.003-0.018
	**Out-of-pocket cost**		–.031		<.001		–0.038 to –0.024		.066			<.001		0.035-0.097
**WTP space estimates**														
	**ASC**		200.153		<.001		154.694-245.612		208.949			<.001		163.235-254.663
	**Doctor level**													
		Ordinary doctors	Reference	Reference		Reference		Reference			Reference		Reference	
		Well-known experts	72.786	<.001		61.445-84.128		49.111			<.001		62.566-35.656	
		General experts	18.684	<.001		9.545-27.823		1.567			.91		29.625-26.491	
	**Hospital level**													
		County-level hospitals	Reference	Reference		Reference		Reference			Reference		Reference	
		Provincial tertiary hospitals	87.429	<.001		74.820-100.039		71.251			<.001		58.043-84.459	
		Municipal-level hospitals	65.553	<.001		54.633-76.472		28.456			.01		50.370-6.542	
	**Consultation format**													
		Text consultation	Reference	Reference		Reference		Reference			Reference		Reference	
		Telephone consultation	9.962	.02		1.277-18.646		0.302			.98		21.572-22.176	
	**Consultation duration**		–1.306		.29		–3.707 to 1.095		1.660			.001		0.639-2.680
	**Waiting time**		–1.948		<.001		–2.566 to –1.330		0.928			<.001		0.532-1.324

^a^WTP: willingness to pay.

^b^OMC: online medical consultation.

^c^ASC: alternative specific constant.

**Table 5:** Comparison of preference, RIS^a^, and WTP^b^ for OMC^c^ in different scenarios.

Attribute and levels			For-Drugs						For-Advice				
			Preference coefficient (*P* value)		RIS (*P* value)		WTP coefficient (*P* value)		Preference coefficient (*P* value)		RIS (*P* value)		WTP coefficient (*P* value)
**Doctor level**					0.179 (<.001)						0.170 (<.001)		
	Ordinary doctors	Reference		Reference		Reference		Reference		Reference		Reference	
	Well-known experts	1.230 (<.001)		N/A^d^		67.127 (<.001)		1.362 (<.001)		N/A		72.786 (<.001)	
	General experts	0.396 (<.001)		N/A		18.496 (<.001)		0.418 (<.001)		N/A		18.684 (<.001)	
**Hospital level**					0.214 (<.001)						0.193 (<.001)		
	County-level hospitals	Reference		Reference		Reference		Reference		Reference		Reference	
	Provincial tertiary hospitals	1.464 (<.001)		N/A		86.542 (<.001)		1.549 (<.001)		N/A		87.429 (<.001)	
	Municipal-level hospitals	0.946 (<.001)		N/A		57.8 (<.001)		1.095 (<.001)		N/A		65.553 (<.001)	
**Consultation format**					N/A						0.029 (.002)		
	Text consultation	Reference		Reference		Reference		Reference		Reference		Reference	
	Telephone consultation	0.131 (.06)		N/A		6.556 (.11)		0.234 (.002)		N/A		9.962 (.02)	
**Consultation duration**			0.029 (<.001)		0.082 (<.001)		–1.105 (.256)		–0.030 (.001)		0.037 (<.001)		–1.306 (.29)
**Waiting time**			–0.003 (<.001)		0.043 (.001)		–3.642 (<.001)		–0.004 (<.001)		0.083 (<.001)		–1.948 (<.001)
**Out-of-pocket cost**			–0.026 (<.001)		0.482 (<.001)		N/A		–0.031 (<.001)		0.487 (<.001)		N/A

^a^RIS: relative importance score.

^b^WTP: willingness to pay.

^c^OMC: online medical consultation.

^d^N/A: not available.

## Discussion

### Principal Findings

This is one of the first studies of the demand by individuals living with overweight or obesity for OMC in China. We identified 2 typical demand scenarios, For-Drugs and For-Advice, and used DCE to compare patient preferences in the 2 scenarios. Our goal is to reveal the different preferences of individuals living with overweight or obesity for OMC in the different demand scenarios.

Our results demonstrated similar preferences for OMC in both scenarios. Participants in both the For-Advice and For-Drugs scenarios preferred doctors with higher-level titles and from higher-level hospitals, with less waiting time and lower cost. In both scenarios, the RIS estimation revealed that cost had the highest influence on participants’ choices (over 48%), followed by hospital level and doctor level. The similarities in patients’ For-Advice and For-Drugs preferences identified key OMC competitive advantages, mainly pricing and providing doctors with higher-level titles and from higher-level hospitals, especially tertiary hospitals.

The DCE results showed that the For-Drugs scenario patients preferred longer consultation time (β=.029; *P*<.001), while For-Advice participants preferred shorter consultation time (β=–.030; *P*=.001). This demand difference has been neglected in previous OMC studies [37]. Combined with our qualitative interviews conducted after the DCE, the time differences suggest that For-Drugs consultations were not deemed urgent by patients, but patients wanted more time to discuss their problems, especially any drug side effects. In our interviews, we found the medication needs of adults living with overweight or obesity mainly fell into 2 categories: one for long-term chronic disease management and one for weight loss, which also has long-term effects. The long-term medication needs of For-Drugs participants explain their preference for longer consultation time.

Our DCE results revealed telephone consultation was preferred in the For-Advice scenario (β_preference_=.0234 and β_WTP_=9.962) compared to the For-Drugs scenario (β_preference_ and β_WTP_ not significant). For-Advice participants viewed consultations as more urgent than For-Drugs patients, with For-Advice patients seeking a quick and direct response to their questions, preferring telephone consultations. We recommend that OMC platforms distinguish the different time preferences and consultation types for users living with overweight or obesity with different demands. For example, it is possible to provide a specific consultation channel to address medication issues where text and longer consultations are provided. Our qualitative interviews also found that adults individuals living with overweight or obesity cared about the long-term effects of medication. We recommend OMC platforms provide long-term consultation service packages for For-Drugs users to achieve better medication adherence management. By comparing the answers of participants from the 2 scenarios in our qualitative interview, we found that compared to drug-use consultations, the preferences for medical advice emphasized urgent responses, with adults living with overweight or obesity seeking quick and direct consultations, which was consistent with our DCE estimation. We recommend the OMC platforms provide more telephone consultations for For-Advice patients, with doctors responding clearly and directly.

The different RIS estimates in the 2 scenarios revealed that in the For-Drugs scenario, consultation duration was relatively more important than waiting time, but in the For-Advice scenario, waiting time was more important than consultation duration. The RIS estimation also revealed that the consultation format was not an attribute that contributed to respondents’ choice in the For-Drugs scenario; however, in the For-Advice scenario, the consultation format contributed 2.9% to the choice made by respondents. RIS estimation verified our explanation that the For-Advice scenario patients needed more urgent and direct consultation. Although high-end medical resources are scarce in China, we recommend the OMC platforms, supported by regulators, allocate more higher-level doctors, short and efficient telephone consultations, satisfying For-Advice users’ preferences and maximizing the use of scarce high-end doctor services.

The WTP estimation showed that respondents in the For-Advice scenario were willing to pay more for doctors with higher-level titles and from higher-level hospitals and telephone consultations, which supports our recommendation that OMC platforms allocate more high-end consultation resources to the For-Advice patients in obesity treatment. Since For-Drugs patients prefer longer consultations, while For-Advice want quick replies, we also recommend doctors schedule long text medication consultations for their For-Drugs patients, but not for their For-Advice patients who prefer telephone consultations. To increase OMC performance, doctors should categorize patients as For-Advice and For-Drugs, with different consultation formats, different mix of telephone and text messaging, and different waiting times. Regulators should allow and encourage OMC platforms to differentiate web-based medical services to maximize the use of medical resources.

### Comparison With Prior Work and Strengths

This study is among the first to study obese individuals’ preferences for OMC, and our findings provided more detailed implications compared with previous studies [38]. Previous studies on OMC neglected the different demands of patients or users [39-41]. By identifying 2 OMC demand scenarios, we drew detailed findings and implications to inform the OMC industry compared to previous studies [40,41]. Second, previous OMC studies lumped together the demands of patients with different diseases, which may cause selection bias. Given the worldwide obesity epidemic, by focusing on obesity, we designed our experiments to obtain specific recommendations for OMC obesity treatment. Third, chronic diseases have become a major global health threat. Obesity is a major chronic disease and is correlated with the causes of many other chronic diseases. Medication is a vital step in the treatment of most chronic diseases. By comparing the preferences of individuals living with overweight or obesity in For-Advice and For-Drugs scenarios, our research findings have implications for other chronic disease treatments. Fourth, the sampling number in previous OMC studies using DCE methods was seldom over 200 patients. By using the quota sampling method, our research recruited 800 respondents nationally following the epidemiological characteristics of China’s adults living with overweight or obesity, which ensured the representativeness of our research.

### Limitations

Our study also has a number of limitations. First, we defined OMC as a paid service without considering free web-based consultations, so our results only apply to paid OMC platforms. Free OMC is not comparable to paid OMC in the range and quality of services, and free OMC uses the service as a point of sale for other services. Separate studies should design surveys for free OMC services. Second, while we noted the popularity of weight-loss drugs, such as semaglutide, our For-Drugs DCE does not provide insights into preferences for any special medicine. Limiting the DCE to a specific weight-loss drug would introduce choice bias, and alternative approaches, such as qualitative interviews, are better suited to research on specific weight-loss drugs. Third, in our experiments, OMC service providers were limited to doctors without considering other professions, such as dietitians and fitness instructors, who were also obesity consultants. Alternative research designs should investigate nonmedical technology interventions in obesity treatment [42]. Finally, we used quota sampling based on the national survey in 2020 to recruit our participants. Future studies should investigate alternative quota databases providing the latest epidemiological characteristics of China’s overweight and individuals living with obesity.

### Future Directions

The future development of OMC depends highly on users’ choices. Not all diseases are suited to OMC and patients with different diseases may have different preferences for web-based consultation. Using DCE, future OMC research should investigate the OMC users’ demand preferences for other diseases. We also recommend more comparative research on the treatment effects between web-based consultation and offline outpatient services.

### Conclusions

Obesity is a unique chronic disease. We identified different For-Drugs and For-Advice demand scenarios in obesity OMC use. Based on the ML model to estimate preferences, WTP, and RIS, we found respondents in both scenarios preferred doctors with higher titles and from higher-level hospitals, less waiting time, and low cost. Consultation duration was relatively more important than waiting time for respondents in the For-Drugs scenario, while shorter waiting time was a more important preference than longer consultation duration in the For-Advice scenario. We also found the group in the For-Advice scenario would pay more for doctors with higher titles and telephone consultations. In the For-Drugs scenario, patient consultation was more important than a quick consultation, while For-Advice patients sought prompt telephone responses. We recommend OMC platforms provide different services catering to the different preferences and WTP for individuals living with obesity. We also recommend doctors differentiate service priorities for For-Advice and For-Drugs patients living with overweight or obesity. Regulators should allow and encourage OMC platforms to differentiate web-based medical services, including pricing, by user need to allocate scarce high-end medical resources efficiently.

## Appendix

Multimedia Appendix 1 Discrete choice experiment explanation of the For-Drugs scenario.

Multimedia Appendix 2 Discrete choice experiment explanation of the For-Advice scenario.

Multimedia Appendix 3 A checklist for discrete choice experiments conducted among Chinese individuals living with obesity.

## Abbreviations

ASC: alternative specific constant
DCE: discrete choice experiment
ISPOR: International Society for Pharmacoeconomics and Outcomes Research
ML: mixed logit
OMC: online medical consultation
RIS: relative importance score
WTP: willingness to pay
