# Supplementary figures and images for: Preferences of Individuals With Obesity for Online Medical Consultation in Different Demand Scenarios: Discrete Choice Experiments
Source: J Med Internet Res. 2024 Nov 27;26:e53140. doi: 10.2196/53140 (PMC11635326; doi:10.2196/53140)

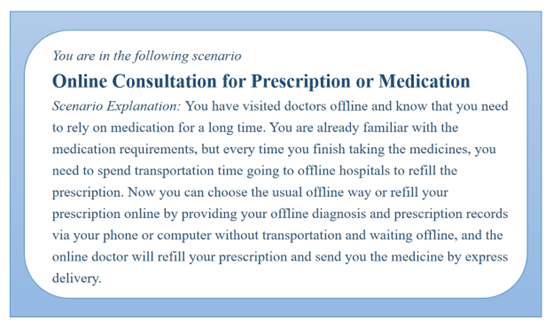

Supplement: Multimedia Appendix 1 [file jmir_v26i1e53140_app1.png]

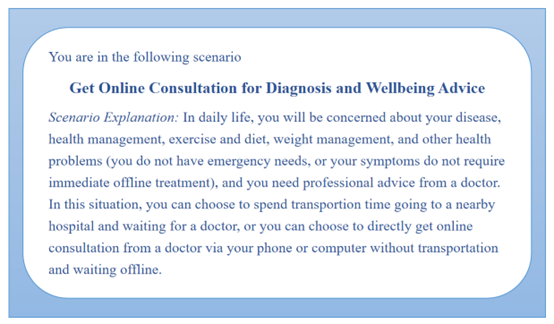

Supplement: Multimedia Appendix 2 [file jmir_v26i1e53140_app2.png]
