# Supplementary material for: Preferences of Individuals With Obesity for Online Medical Consultation in Different Demand Scenarios: Discrete Choice Experiments
Source: J Med Internet Res. 2024 Nov 27;26:e53140. doi: 10.2196/53140 (PMC11635326; doi:10.2196/53140)
Supplement: Multimedia Appendix 3 [file jmir_v26i1e53140_app3.pdf]

A Checklist for Discrete Choice Experiments Conducted among Chinese living with Obesity [a]

Items to Check		Check
**1. Was a well-defined research question stated and is conjoint analysis an appropriate method for answering it?**		√
	1.1 Were a well-defined research question and a testable hypothesis articulated?	To assess and compare the preferences for For-Drug and For-Advice OMC among individuals living with obesity in China.
	1.2 Was the study perspective described, and was the study placed in a particular decision-making or policy context?	To provide suggestions for OMC industries and regulation departments.
	1.3 What is the rationale for using conjoint analysis to answer the research question?	➢ Capturing Complex Preferences: Obesity patients have complex preferences for OMC that depend on various factors. DCE allows for the modeling of these multi-faceted preferences by presenting respondents with a series of hypothetical choices and measuring trade-offs between attributes.<br>➢ Simulating Realistic Decision Making: DCE mimics real-world decision-making by asking respondents to choose between different options, each described by several attributes at different levels. This simulates the actual choice scenario patients face when selecting an OMC service, providing insights into their preferences under realistic conditions.<br>➢ Eliciting Willingness to Pay (WTP): DCE can

Items to Check		Check
		estimate WTP for each attribute, which is essential for understanding the value patients place on different aspects of OMC and for pricing strategies.<br>➢ Comparing Different Demand Scenarios: Our study aims to compare preferences between two distinct scenario groups (For-Drug and For-Advice). DCE is well-suited for this comparative analysis, as it can incorporate different scenarios into the design, allowing for direct comparison of preferences across these groups.
**2. Was the choice of attributes and levels supported by evidence?**		√
	2.1 Was attribute identification supported by evidence (literature reviews, focus groups, or other scientific methods)?	Supported by a combination of literature reviews, qualitative research, and focus groups with obese or overweight patients and consultations with obesity experts.
	2.2 Was attribute selection justified and consistent with theory?	The attributes were grounded in health economics literature and aligned with the principles of utility theory, which underpins the DCE method.
	2.3 Was level selection for each attribute justified by the evidence and consistent with the study perspective and hypothesis?	The level selection for each attribute was justified by evidence from the literature and qualitative research, ensuring that they were representative of the real-world choices faced by obese patients.

Items to Check		Check
		The levels were also consistent with the study's perspective and hypotheses, allowing for a meaningful examination of patient preferences across different aspects of OMC.
**3. Was the construction of tasks appropriate?**		√
	3.1 Was the number of attributes in each conjoint task justified (that is, full or partial profile)?	Partial profile.
	3.2 Was the number of profiles in each conjoint task justified?	Yes, the number of profiles in each conjoint task was justified, providing an adequate level of information while minimizing respondent burden.
	3.3 Was (should) an opt-out or a status-quo alternative (be) included?	We provided opt-out by dual-response choices.
**4. Was the choice of experimental design justified and evaluated?**		√
	4.1 Was the choice of experimental design justified? Were alternative experimental designs considered?	D-efficient design was employed to ensure the most precise parameter estimates with a manageable number of choice sets.<br>Alternative designs were considered, but the chosen design optimally balanced the trade-off between information richness, experimental efficiency, and respondent burden.
	4.2 Were the properties of the experimental design evaluated?	The properties of the experimental design were evaluated to ensure it met the requirements for precision and reliability. This included assessing the design's D-efficiency and orthogonality to

Items to Check		Check
		guarantee that the chosen design could effectively estimate the main and interaction effects of the attributes on choice behavior.
	4.3 Was the number of conjoint tasks included in the data-collection instrument appropriate?	Yes.
**5. Were preferences elicited appropriately, given the research question?**		√
	5.1 Was there sufficient motivation and explanation of conjoint tasks?	Yes, participants were provided with clear motivation and comprehensive explanations for the conjoint tasks. This included an introduction to the DCE methodology and the purpose of the study, ensuring that respondents understood the context and could provide informed preferences.
	5.2 Was an appropriate elicitation format (that is, rating, ranking, or choice) used? Did (should) the elicitation format allow for indifference?	Yes, an appropriate elicitation format was used, specifically a choice-based format, which is well-suited for DCE and allows for the expression of indifference by providing an opt-out option by dual-response choice design.
	5.3 In addition to preference elicitation, did the conjoint tasks include other qualifying questions (for example, strength of preference, confidence in response, and other methods)?	Several participants interviews were conducted in the pilot experiments and after the experiments.
**6. Was the data collection instrument designed appropriately?**		√
	6.1 Was appropriate respondent information collected (such as sociodemographic, attitudinal, health history or status, and treatment experience)?	Yes.
	6.2 Were the attributes and levels defined, and was any	Yes.

Items to Check		Check
	contextual information provided?	
	6.3 Was the level of burden of the data-collection instrument appropriate? Were respondents encouraged and motivated?	Yes, the level of burden was appropriate, ensuring the data-collection instrument was not overly demanding for respondents. The survey was designed to be concise and engaging. Respondents were encouraged and motivated through clear communication of the study's importance and by offering incentives for completion, which helped maintain high response rates and data quality.
**7. Was the data-collection plan appropriate?**		√
	7.1 Was the sampling strategy justified (for example, sample size, stratification, and recruitment)?	Yes.
	7.2 Was the mode of administration justified and appropriate (for example, face-to-face, pen-and-paper, web-based)?	Online experiment and offline support are combined.
	7.3 Were ethical considerations addressed (for example, recruitment, information and/or consent, compensation)?	Yes.
**8. Were statistical analyses and model estimations appropriate?**		√
	8.1 Were respondent characteristics examined and tested?	Yes.
	8.2 Was the quality of the responses examined (for example, rationality, validity, reliability)?	The quality of the responses was rigorously examined to ensure rationality, validity, and reliability. This included checks for logical consistency in choices, the use of statistical methods to validate the responses against the

Items to Check		Check
		theoretical model, and pilot testing to confirm that the DCE produced reliable preference measures.
	8.3 Was model estimation conducted appropriately? Were issues of clustering and subgroups handled appropriately?	Model estimation was conducted appropriately using mixed logit models, which are suitable for handling the complexity of DCE data. Issues of clustering and potential subgroups were addressed by incorporating random effects in the model, allowing for heterogeneity in preferences across different segments of the respondent population.
**9. Were the results and conclusions valid?**		√
	9.1 Did study results reflect testable hypotheses and account for statistical uncertainty?	The study's results were based on testable hypotheses derived from the DCE data, and statistical uncertainty was accounted for through confidence intervals and significance testing. This approach ensured that the findings were both robust and generalizable.
	9.2 Were study conclusions supported by the evidence and compared with existing findings in the literature?	Yes.
	9.3 Were study limitations and generalizability adequately discussed?	Yes.
**10. Was the study presentation clear, concise, and complete?**		√
	10.1 Was study importance and research context adequately motivated?	The study importance and research context were adequately motivated, providing respondents

Items to Check		Check
		with a clear understanding of the relevance and purpose of the research. This motivation is crucial for engaging participants and ensuring they provide thoughtful and meaningful responses.
	10.2 Were the study data-collection instrument and methods described?	Yes.
	10.3 Were the study implications clearly stated and understandable to a wide audience?	Yes.

[a] The checklist is derived from the scientific report by ISPOR (Bridges et al., 2011).
